# Tropical *Drosophila ananassae* of wet-dry seasons show cross resistance to heat, drought and starvation

**DOI:** 10.1242/bio.029728

**Published:** 2017-11-15

**Authors:** Chanderkala Lambhod, Ankita Pathak, Ashok K. Munjal, Ravi Parkash

**Affiliations:** 1 Department of Genetics, Maharshi Dayanand University, Rohtak 124001, India; 2 Department of Biochemistry and Genetics, Barkatullah University, Bhopal 462026, India

**Keywords:** Seasonal adaptations, Plastic changes, Stress-related traits, Energy metabolites, Cross protection, *Drosophila ananassae*

## Abstract

Plastic responses to multiple environmental stressors in wet or dry seasonal populations of tropical *Drosophila* species have received less attention. We tested plastic effects of heat hardening, acclimation to drought or starvation, and changes in trehalose, proline and body lipids in *Drosophila ananassae* flies reared under wet or dry season-specific conditions. Wet season flies revealed significant increase in heat knockdown, starvation resistance and body lipids after heat hardening. However, accumulation of proline was observed only after desiccation acclimation of dry season flies while wet season flies elicited no proline but trehalose only. Therefore, drought-induced proline can be a marker metabolite for dry-season flies. Further, partial utilization of proline and trehalose under heat hardening reflects their possible thermoprotective effects. Heat hardening elicited cross-protection to starvation stress. Stressor-specific accumulation or utilization as well as rates of metabolic change for each energy metabolite were significantly higher in wet-season flies than dry-season flies. Energy metabolite changes due to inter-related stressors (heat versus desiccation or starvation) resulted in possible maintenance of energetic homeostasis in wet- or dry-season flies. Thus, low or high humidity-induced plastic changes in energy metabolites can provide cross-protection to seasonally varying climatic stressors.

## Abstract (precis)

**Summary:** In the tropical *Drosophila ananassae*, low or high humidity-induced plastic changes in energy metabolites provide cross-protection to seasonally varying climatic stressors.

## INTRODUCTION

In ectothermic organisms living under seasonally varying stressful climatic conditions, maintenance of energetic homeostasis is a major challenge (Lee, 2010). Stressor-induced changes in the level of energy metabolites can limit survival of insects under harsh climatic conditions (Rosendale et al., 2017). In some ectothermic organisms from temperate regions, single or multiple bouts of cold- or drought-induced plastic changes have shown reduction of certain energy metabolites (Overgaard et al., 2007; Kostal et al., 2011; Colinet et al., 2012; Teets et al., 2011, 2012). In tropical insect taxa, seasonal changes involve major shifts in relative humidity and temperature (Tauber et al., 1998; Chown et al., 2011). Changes in cuticular components are associated with altered levels of drought resistance in seasonal (dry or wet) populations of *Anopheles gambiae* from the African region Sahel (Wagoner et al., 2014); and in some tropical *Drosophila* species (Parkash et al., 2009; Parkash and Ranga, 2014). However, energetic consequences of humidity-triggered plastic changes to multiple stressors have received less attention in tropical insects.

In temperate regions, ectothermic organisms encounter a greater range of colder environments (freezing to mild/warm) which affect their acclimatization to improve survival under unfavorable conditions (Angilletta, 2009). Stressor-specific utilization of carbohydrates, body lipids and proteins has been demonstrated in diverse insect taxa such as mosquito (*Culex pipiens*, Benoit et al., 2010); American dog tick (*Dermacentor variabilis*, Rosendale et al., 2017); in larvae of Antarctic midge (*Belgica antartica*, Benoit et al., 2007; Teets et al., 2012); in xeric and mesic *Drosophila* species (Marron et al., 2003); and in *Drosophila melanogaster* (Kostal et al., 2011; Colinet et al., 2012). Energy depletion under single or multiple bouts of cold or desiccation stress have shown species-specific as well as stressor-specific differences in the percent consumption of metabolite reserves. For example, in the Antarctic midge (*Belgica Antarctica*), repeated exposure of cold or dehydration stress resulted in ∼67% loss of carbohydrate content (Teets et al., 2011, 2012). In the latter study, 27% decrease in body lipids was evident when the larvae of Antarctic midge were subjected to prolonged dehydration for 10 days while repeated exposure of dehydration stress exhibited no decline in the level of body lipids. A study on the effects of single bout versus repeated bouts (3, 6 or 12 cycles) of dehydration in American dog tick showed that single bout led to significant decrease in the level of protein and body lipids but not in the glycogen level, while effects of repeated bouts significantly decreased only body lipids (Rosendale et al., 2017). Therefore, energetic consequences of dehydration stress seem complex and could vary between diverse insect taxa.

Freeze-tolerant, freeze-avoiding and chill-susceptible organisms undergo stressor-induced plastic changes in metabolites such as sugars and polyols (Overgaard et al., 2007; Michaud et al., 2008; Kostal et al., 2011; Colinet et al., 2012). However, in the freeze-tolerant gall fly *Eurosta solidaginis*, cold-induced energy metabolites include glycerol and sorbitol (Lee, 2010). Utilization of body lipids has been associated with drought-induced changes in *Culex pipiens* (Benoit et al., 2010) and in American dog tick (Rosendale et al., 2017). As compared to many studies on sugars and polyols, changes in free amino acids accumulated in response to different climatic stressors have been investigated in few insect taxa (Fields et al., 1998; Misener et al., 2001); and in arthropod (Issartel et al., 2005). In insects, accumulation of proline and trehalose has been associated with cryoprotective function (Kostal et al., 2011). However, partial oxidation of proline, trehalose and glycogen during insect flight reflects their major role as metabolic fuels. Although, energy yield due to oxidation of lipids (0.65 mol ATP) is slightly higher than proline (0.52 mol ATP), the latter is an important energy source due its higher water solubility, resynthesis of proline from alanine and acetyl Co-A, and it does not require any carrier molecule (Bursell, 1981; Gäde and Auerswald, 2002). Thus, proline and trehalose (which occur abundantly in insect's hemolymph) can serve as metabolic fuels. In insects, the trehalose-trehalase system is also known to provide metabolic energy (Chapman, 1998). In *Belgica Antarctica*, exogenous supply of trehalose significantly improved survival to cold and to heat stress (Benoit et al., 2009). Therefore, multiple stressor-induced plastic changes are likely to involve shared physiological mechanisms.

In the tropics, seasonal changes in humidity conditions play a major role in affecting morphological, physiological and life history traits in insects (Tauber et al., 1986, 1998). For example, the role of humidity has been demonstrated for dry-season-induced diapause in some tropical insects (Pires et al., 2000; Seymour and Jones, 2000); increased desiccation resistance due to low humidity developmental acclimation in *D. leontia* (Parkash and Ranga, 2014); and due to effect of humidity acclimation on heat resistance in adult flies of *D. simulans* (Bubliy et al., 2013). Further, another study on tropical *D. jambulina* has shown the effect of low- versus high-humidity acclimation on mating-related traits of darker and lighter morphs consistent with melanism-desiccation hypothesis (Parkash et al., 2009). This study has shown that the frequencies of melanic and non-melanic morphs of *D. jambulina* are driven by humidity changes and not due to thermal conditions (Parkash et al., 2009). Wet or dry seasonal morphs of *Anopheles gambiae* (from the African locality Sahel) revealed changes in the level of three amino acids (phenylalanine, tyrosine and valine) which are likely to impact cuticular permeability (Hidalgo et al., 2014); and in the amount of cuticular hydrocarbons (Wagoner et al., 2014). Thus, for tropical insect taxa of wet–dry seasons, there is a need to assess acclimatization potential to multiple stressors (Tauber et al., 1998; Hoffmann, 2010; Chown et al., 2011).

Seasonality in the tropical regions is associated with significant changes in water stress from rainy to dry season within a year, rather than with thermal stress. On the Indian subcontinent, there are contrasting seasonal patterns of ambient relative humidity (RH) (80±5% RH during monsoon but 40±6% RH during autumn; www.tropmet.res.in). In Southeast Asia, changes in relative humidity associated with reduced patterns of rainfall due to El Niño and climate warming are likely to increase drier conditions affecting survival of tropical insect taxa (www.skymetweather.com; www.imd.gov.in). In the present work, we assessed season-specific and sex-specific plastic changes in stress-related traits. We also investigated changes in the levels of three energy metabolites in the tropical *D. ananassae* which is characterized by low desiccation resistance. Wild-caught flies of *D. ananassae* from wet (monsoon) and dry (autumn) seasons were reared under season-specific simulated growth conditions and flies were tested for basal and induced level of resistance to heat, desiccation and starvation resistance. For each stressor, we tested cross resistance for other two stressors. Further, we investigated patterns of changes (accumulation and/or utilization) for each of the three metabolic fuels due to plastic changes. For control as well as acclimated flies, changes in energy metabolites were also estimated. The rates of change in the levels of three energy metabolites were assessed in three replicates of multiple groups of flies subjected to different time durations of heat hardening, desiccation or starvation acclimation. We aim to find stressor-induced plastic changes for possible maintenance of energetic homeostasis in the tropical *D. ananassae* from wet or dry seasons.

## RESULTS

### Seasonal differences in stress resistance and energy metabolites

Data on seasonal differences (wet versus dry) for six traits of *D. ananassae* flies reared under season-specific simulated conditions are shown in Table 1. For heat knockdown, starvation resistance and body lipids, wet season flies revealed significantly higher trait values as compared with dry season flies (Table 1). For dry season flies, desiccation resistance and the amount of trehalose and proline were significantly higher. For all the traits, season-specific differences were significant for both the sexes (*P*<0.001; Table 1).

**Table 1.**
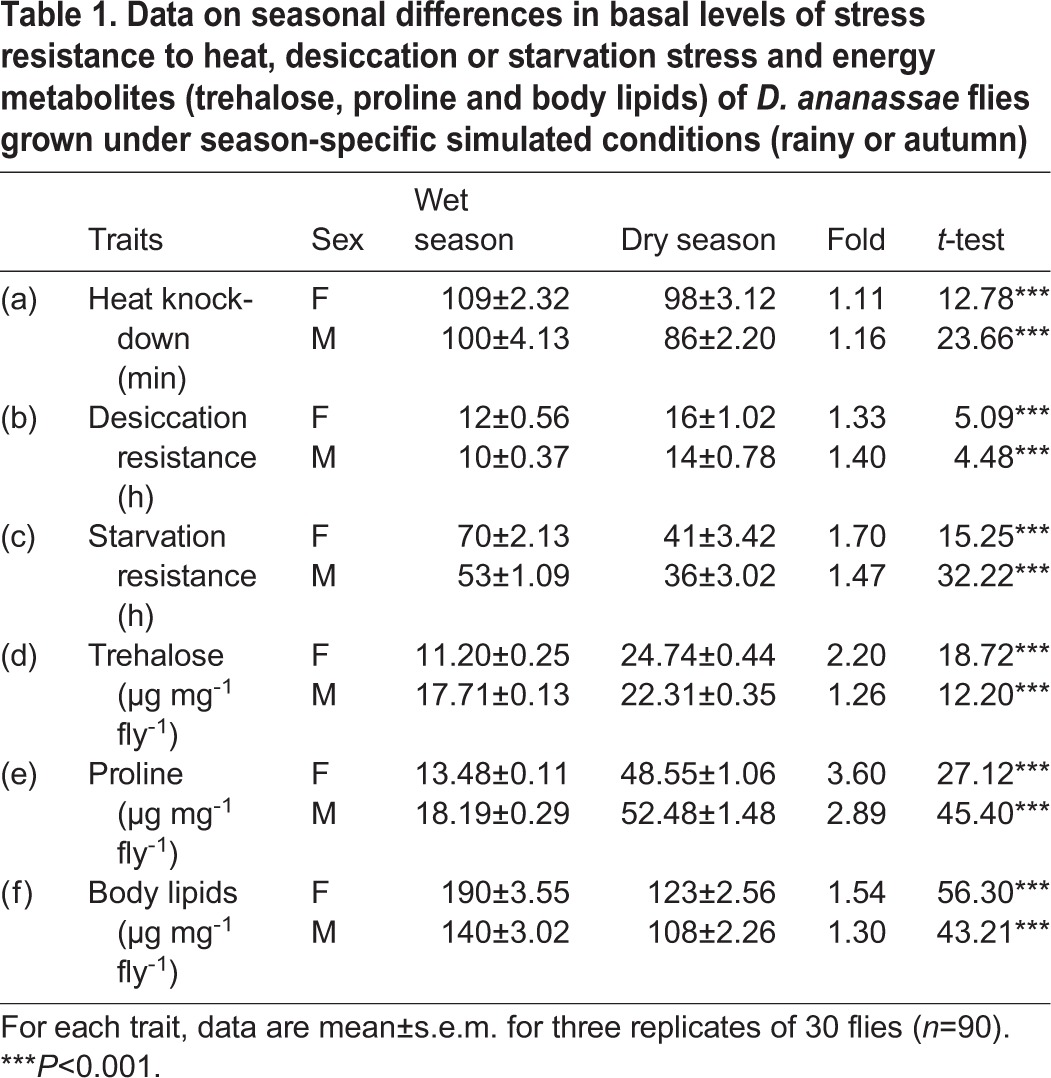
**Data on seasonal differences in basal levels of stress resistance to heat, desiccation or starvation stress and energy metabolites (trehalose, proline and body lipids) of *D. ananassae* flies grown under season-specific simulated conditions (rainy or autumn)**

In Table S1, we have shown results of ANOVA for three stress-related traits (heat, drought or starvation) with respect to three variables (control versus acclimated; sexes and seasons) in *D. ananassae* flies reared under wet or dry season-specific conditions. For all the three stress-related traits, we found highly significant differences (*P*<0.001) across sexes, seasons and due to acclimation as well as due to their respective interactions (Table S1). Similarly the results of ANOVA on three energy metabolites are shown in Table S2 which also showed significant differences (*P*<0.001) for all the variables.

### Plastic changes in heat knockdown of wet versus dry season flies

**Fig. 1.**
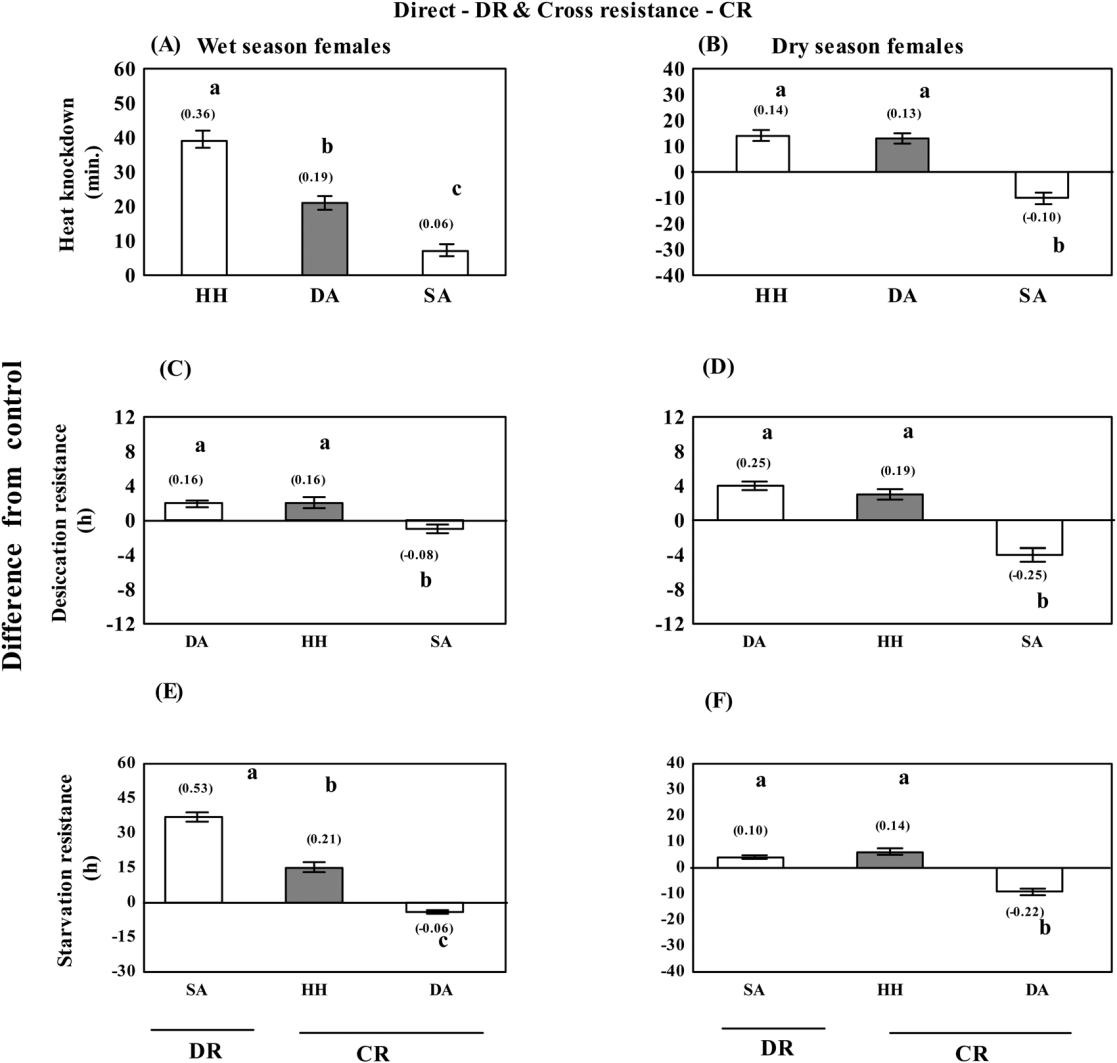
**Seasonal changes in hardening/acclimation effects (direct as well as cross tolerance) to three stressors (heat, desiccation or starvation).** Plastic changes (acclimated trait value–control) in heat knockdown (minutes) desiccation resistance (h); starvation resistance (h) as a consequence of hardening/acclimation (direct effect) and due to cross tolerance effects in female flies of *D. ananassae* of wet or dry season. Relative acclimation capacity (RAC) value is shown on top of each bar. Different letters above the bars represent significant differences (*P*<0.01, Tukey's test). Error bars indicate mean±s.e.m.

For heat knockdown, data on absolute hardening capacity (acclimated-control values) due to direct hardening and cross tolerance effects due to desiccation or starvation acclimation are illustrated in Fig. 1A,B. There was significant increase in heat knockdown due to heat hardening; and cross resistance effect of desiccation in wet season female flies (Fig. 1A). However, lesser increase in heat knockdown was observed in starvation acclimated flies of wet season (Fig. 1A). In contrast, in dry season flies, heat knockdown duration decreased as a consequences of cross tolerance effect due to starvation acclimation (Fig. 1B). Plastic changes in heat knockdown of dry season flies due to direct heat hardening and cross tolerance effect after desiccation acclimation were 50 to 60% lower (Fig. 1B). Therefore, direct effect due to heat hardening and cross tolerance effects significantly improved heat resistance of wet season flies as compared with dry season flies (Fig. 1A,B).

### Season-specific plastic changes in resistance to desiccation or starvation

**Fig. 2.**
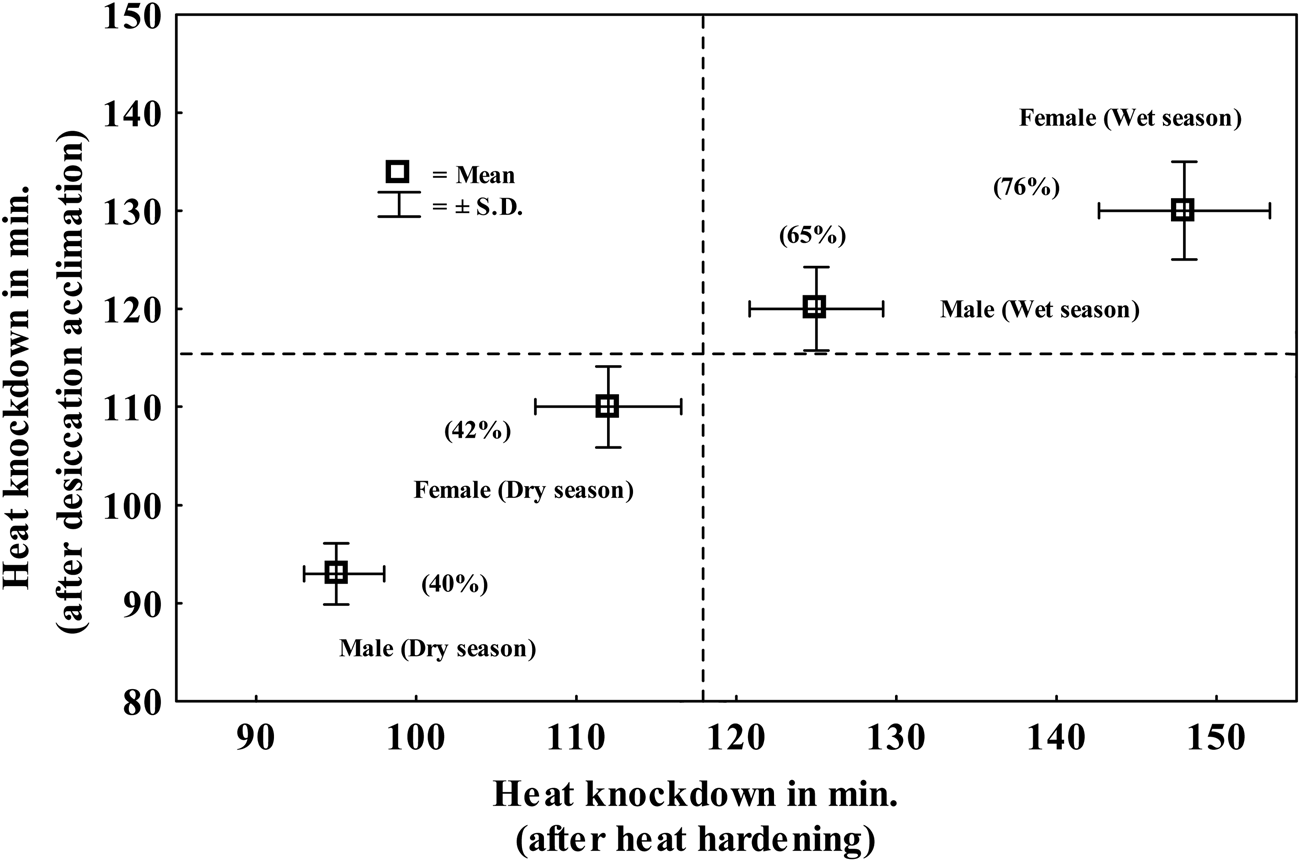
**Relationship between plastic changes in heat knockdown due to direct effect of heat hardening versus cross tolerance effect due to desiccation acclimation.** Percent changes in energy metabolite per fly due to plastic changes are indicated in parentheses.

Wet season flies exhibited lower acclimation effects due to desiccation acclimation, as well as cross tolerance of heat-hardened flies (Fig. 1C) as compared with dry season flies (Fig. 1D). There was a trade-off between desiccation and starvation resistance. We observed twofold reduction in desiccation resistance of starvation acclimated flies of dry season as compared with wet season. For starvation resistance, wet season flies exhibited a significantly higher effect of direct starvation acclimation; and cross tolerance due to heat hardening as compared with dry season flies (Fig. 1E,F). In case of desiccation-acclimated flies, there was greater reduction in starvation resistance of dry season flies as compared with wet season flies. Finally, for seasonal changes in heat knockdown, we found a positive relationship between direct heat hardening and cross tolerance due to desiccation acclimation (Fig. 2). Thus, significant increase in heat resistance of wet season flies results due to plastic effects of heat and drought (Fig. 2).

### Assessment of relative hardening/acclimation capacity

Results of relative acclimation capacity (RAC) for direct as well as cross-tolerance plastic effects for three stressors are shown in Fig. 1. In wet season flies, direct acclimation effects were maximum for starvation acclimation followed by heat hardening; however, direct effect of desiccation acclimation was higher for dry season flies as compared with wet season flies. RAC values for cross-tolerance effects [starvation acclimation (SA) on heat knockdown (HK); desiccation acclimation (DA) on HK and DA on heat hardening (HH)] were quite similar, i.e. RAC=∼0.20 (Fig. 1). These observations suggest that RAC values for direct acclimation/hardening are stressor-specific while cross tolerance effects could be conserved for a species. This assumption needs further verification for its generality among *Drosophila* species. Further, we observed a trade-off between RAC values of cross-tolerance effects of DA versus SA but such effects were significantly higher for dry season flies (Fig. 1C-F). Finally, cross-tolerance effect of SA on heat knockdown was positive in wet season flies but there was a trade-off (SA on HK) in dry season flies (Fig. 1A,B).

### Accumulation and utilization of energy metabolites

A comparison of changes in each of the three energy metabolites as a consequence of heat hardening or acclimation to desiccation or starvation of wet or dry season flies are given in Table 2. For body lipids, complementary changes are evident for accumulation due to heat hardening and utilization under starvation stress. For trehalose, desiccation acclimation and heat hardening revealed inter-related changes for flies of both the seasons. Finally, for proline, accumulation and utilization due to desiccation acclimation and heat stress are evident for dry season flies only (Table 2).

**Table 2.**
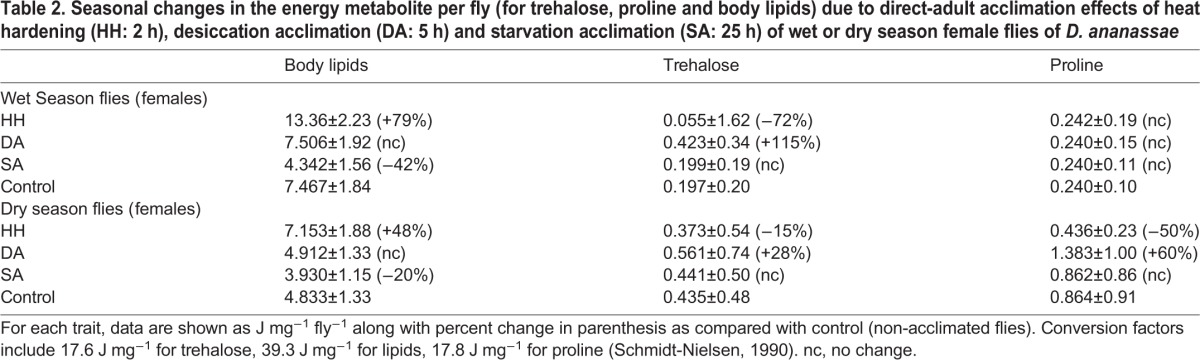
**Seasonal changes in the energy metabolite per fly (for trehalose, proline and body lipids) due to direct-adult acclimation effects of heat hardening (HH: 2 h), desiccation acclimation (DA: 5 h) and starvation acclimation (SA: 25 h) of wet or dry season female flies of *D. ananassae***

### Stressor-specific rates of change in energy metabolites

We found significant seasonal differences in stressor-specific (heat, drought or starvation) changes (accumulation or utilization) in the levels of each of the three energy metabolites as a function of different durations of hardening/acclimation of wet- or dry-season flies (Table 3). For body lipids and trehalose, the rates of change were significantly higher for wet season flies as compared with dry season flies. Sex-specific differences in the rates of metabolite change were observed only in the wet season flies (Table 3). Further, changes in the level of proline were found only in dry season flies.

**Table 3.**
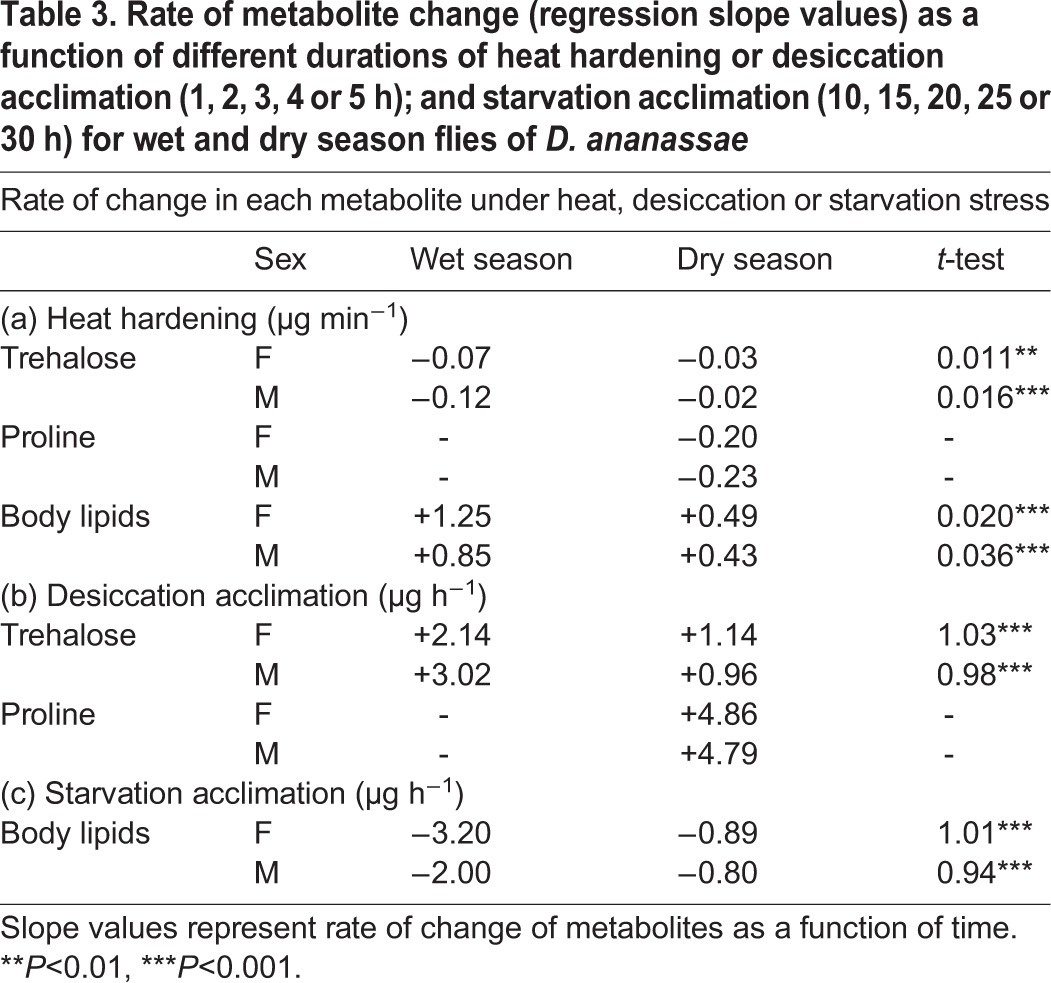
**Rate of metabolite change (regression slope values) as a function of different durations of heat hardening or desiccation acclimation (1, 2, 3, 4 or 5 h); and starvation acclimation (10, 15, 20, 25 or 30 h) for wet and dry season flies of *D. ananassae***

### Energy metabolite mediated cross-protection

**Fig. 3.**
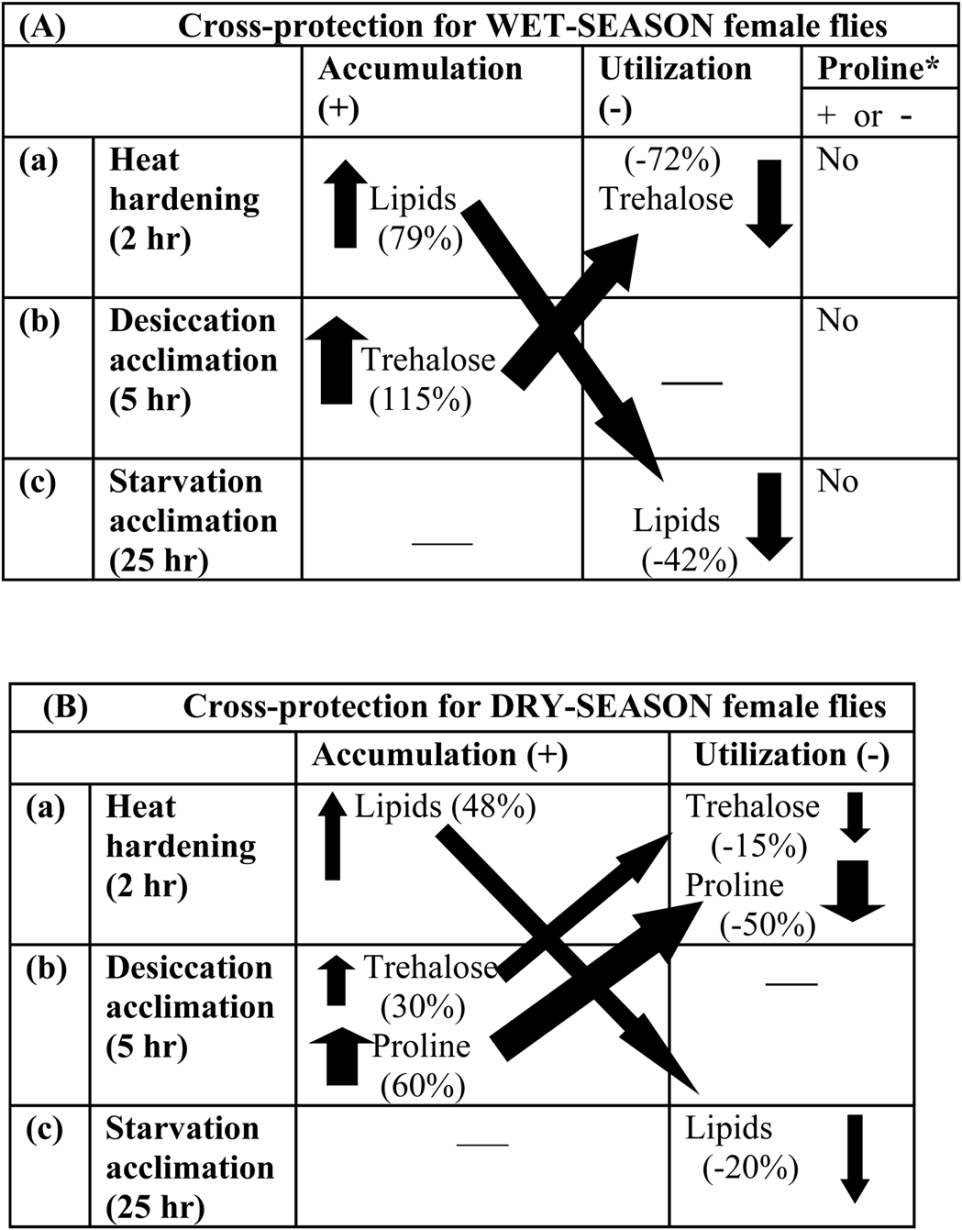
**Schematic representation of stressor-induced (heat, drought or starvation) plastic changes (accumulation and utilization) of three energy metabolites (trehalose, proline and body lipids) in *D. ananassae* female flies of wet or dry season.** (A) Wet, (B) dry season. Slanted arrows depict cross-protection effects. *Lack of changes in the proline content in the wet season female flies.

In *D. ananassae* flies reared under wet or dry conditions, we found significant differences in the stressor-specific levels of accumulation and utilization of trehalose, proline and body lipids (Fig. 3). This schematic diagram shows that body lipids increase due to heat hardening, i.e. ∼79% in wet season flies as compared with 48% for dry season flies. There is cross-protection between accumulation of body lipids due to heat hardening and utilization under starvation. In contrast, proline was elicited by desiccation-acclimated flies of dry season only. However, desiccation-acclimated wet-season flies accumulated 115% more trehalose than control flies. Further, heat hardening of wet season flies utilized 50% of the accumulated trehalose. For dry season flies, desiccation-induced plastic changes accumulated only 30% more trehalose but 60% more amount of proline which were partially utilized under heat hardening. Hence, plastic changes in three energy metabolites (trehalose, proline and body lipids) are consistent with cross-protection between three stressors (heat, drought and starvation).

## DISCUSSION

We observed humidity-driven significant plastic changes in the stress-related traits (heat knockdown, and resistance to desiccation or starvation); and three energy metabolites (proline, trehalose and body lipids) in *D. ananassae* flies reared under wet or dry season-specific simulated conditions. Basal levels of stress resistance and energy metabolites differ significantly due to developmental acclimation. The effects of heat hardening and desiccation acclimation significantly improved heat knockdown in wet season flies. Further, heat hardening also increased starvation resistance in wet season flies. However, dry season flies showed higher levels of proline as well as desiccation resistance but a lower amount of trehalose. Therefore, proline can be considered as a marker metabolite because accumulation of proline was evident only in drought acclimated flies of dry season. Stressor-specific changes in energy metabolites per fly support cross-protection between heat hardening and desiccation or starvation resistance. Thus, relative humidity (wet or dry) induced plastic changes in three energy metabolites are consistent with energetic homeostasis.

### Seasonal differences in cross-tolerance effects

In the present work, we examined cross-tolerance to multiple stressors after developmental and adult acclimation effects of wet or dry conditions. In heat-hardened flies (of wet or dry seasons), cross-tolerance effects on desiccation was higher for dry- than wet-season flies. However, such cross-tolerance effect on starvation tolerance was more in wet- than dry-season flies (Fig. 1). In contrast, in starvation acclimated flies cross-tolerance effects on heat resistance revealed season-specific contrasting differences, i.e. a positive effect in wet season flies (Fig. 1A) but a negative effect in dry season flies (Fig. 1B). Therefore, cross-tolerance effects of starvation acclimated flies on heat knockdown differ across seasons.

Based on genetic effects, a previous study has shown lack of trade-off between resistance to heat and other stress-related traits in *D. melanogaster* (Williams et al., 2012). Genetic and plastic effects of multiple stressors seem to differ in impacting heat tolerance but this assumption needs further analysis. In *D. ananassae*, cross-tolerance has effects in two cases: (a) plastic response of starvation acclimation on desiccation resistance (Fig. 1C,D); and (b) effect of desiccation acclimation on starvation resistance showed trade-off with greater effect in dry than wet season flies (Fig. 1E,F). These observations are consistent with a trade-off between resistance to desiccation or starvation in geographical populations of some tropical drosophilids on the Indian subcontinents (Parkash and Munjal, 1999), and in the American dog tick (Rosendale et al., 2017). Thus, we find similarity between plastic and genetic effects for desiccation versus starvation resistance in geographical as well as seasonal populations of tropical *Drosophila* species.

### Stressor-specific relative acclimation capacity differ across wet-dry seasonal flies

Relative acclimation capacity (RAC) is a quantitative measure to compare stressor-induced plastic changes across species as well as within species, i.e. seasonal or geographical populations of ectothermic organisms (Kellett et al., 2005). The magnitude of RAC for heat knockdown has been found to be similar (RAC=0.25) in interspecific comparisons of four species each of *melanogaster* species group and montium species group (Kellett et al., 2005). In contrast, *D. melanogaster* flies reared at 18, 25 and 28°C showed variable effects of thermal developmental acclimation on heat knockdown, i.e. RAC ∼0.35 for 18 or 25°C reared flies but no RAC effect for 28°C flies (Cavicchi et al., 1995). Further, geographical populations of *D. melanogaster* from east coast of Australia revealed RAC values in the range of 0.13 to 0.58 for heat knockdown (Sgrò et al., 2010).

We are not aware of studies related to assessment of quantitative differences in stressor-specific relative acclimation capacity in seasonally varying populations of diverse insect taxa. In the present work, we found significant season-specific (wet or dry) and stressor-specific differences in relative acclimation capacity (Fig. 1). For direct acclimation effects, RAC values were in the order of SA=0.53>HH=0.36>DA=0.25. In contrast, cross-tolerance RAC values were quite similar (RAC=∼0.20) for HH on SR, DA on HK (wet season flies) and HH on DR (dry season flies). The generality of such observations need further studies. Another interesting observation was cross-tolerance effect of starvation acclimation on heat knockdown, i.e. a positive effect in wet season flies but a negative relationship (trade-off) in dry season flies. We found season-specific and stressor-specific differences in RAC effects in low- versus high-humidity reared flies of *D. ananassae*.

### Cross-protection through inter-related plastic changes in metabolic fuels

In wild habitats, colder or warmer environments are coupled with wet or dry or possible starvation conditions; and insects are likely to evolve cross-protection mechanisms to multiple stressors. It is known that cold stress with coupled drought level is able to elicit multifunctional colligative solutes (sugars, polyols, proline as free amino acids). Possible inter-related modulatory changes (cross-protection) in the accumulation and utilization of metabolic fuels could favor energetic homeostasis. For example, a previous study has shown drought stress induced a higher level of proline but exhibited its utilization under cold stress in winter population of *D. immigrans* (Tamang et al., 2017).

In the present work, we observed cross-protection between heat hardening and two coupled stressors, i.e. drought or starvation. We found inter-related changes in three metabolic fuels both for wet or dry season flies of *D. ananassae*. First, inter-relationship between heat hardening-induced accumulation of body lipids and its consumption in starvation-acclimated flies was evident for both the seasons. However, body lipid changes were 60% higher for wet season flies as compared with dry season. Such observations are consistent with season-specific differences in starvation resistance levels due to developmental and adult acclimation. Secondly, inter-related metabolic changes due to heat hardening or desiccation acclimation involved accumulation and utilization of proline only in dry season flies. Thirdly, we observed fourfold higher (∼115%) accumulation of trehalose in wet season flies as compared to dry season flies. Trehalose as osmoprotectant is known to counter the detrimental effects of drought stress on cellular membranes and cellular proteins. Therefore, plastic changes in metabolic fuels due to multiple stressors seem to involve inter-related compensatory mechanisms to cope with the season-specific wet or dry conditions.

### Proline provides a link between resistance to desiccation and heat in dry season flies

Some studies have suggested a thermoprotective role of proline to heat stress (Malmendal et al., 2006; Szabados and Savouré, 2009). First, association between heat and proline has been observed in beetle *Alphitobius diaperinus* maintained at 28°C because the proline amount constituted 50% out of a pool of 16 amino acids (Renault et al., 2006). Second, heat-hardened *D. melanogaster* adults had elevated levels of alanine (a product of proline oxidation) during the recovery period (Malmendal et al., 2006). Third, thermoprotective and osmoprotective roles of proline have been suggested in some plant taxa (Szabados and Savouré, 2009); and in soil bacterium *Rhizobium etli* (Reina-Bueno et al., 2012). However, such dual functions of proline in diverse insect taxa have received less attention.

In the present work, drought-induced accumulation of proline is associated with acclimatization of *D. ananassae* to drier conditions. *D. ananassae* flies (reared under dry season condition) exhibited significant accumulation of proline but a lower level of trehalose. However, *D. ananassae* flies reared under wet condition did not elicit proline at all despite accumulation of a significant level of trehalose (Fig. 3). Further, drought-induced plastic changes in proline have been observed both for cold-adapted *D. immigrans* (Tamang et al., 2017) and in warm-adapted *Z. indianus* (Kalra et al., 2017). These studies suggest a possible role of proline as osmoprotectant in drosophilids reared under warmer and drier conditions. For *D. ananassae*, we observed partial utilization of proline when dry season flies were subjected to heat hardening (Fig. 3), but we did not examine possible elevation of alanine due to proline oxidation. Further studies are needed to analyze osmo- as well as thermoprotective roles of proline in seasonally varying wild populations of different warm-adapted drosophilids.

### Conclusions

We observed complementary metabolic changes in accumulation and utilization of trehalose and proline in response to desiccation and heat stress in wet- or dry-season flies. Accumulation of proline occurred only in dry season flies in response to desiccation acclimation. We observed variable levels of trehalose in wet- versus dry-season flies. However, heat hardening resulted in partial utilization of trehalose in wet season flies but both proline and trehalose in dry season flies. Thus, proline could be a marker metabolite for dry season flies. Inter-related changes due to heat hardening and starvation acclimation involved accumulation and utilization of body lipids. Finally, we assessed energy metabolite changes per fly in control versus flies hardened/acclimated to heat or drought or starvation. For both the seasons (wet or dry) and sexes, heat hardening increased energy metabolite per fly due to buildup of body lipids which were used during starvation. Thus, energy metabolite changes due to stressors resulted in cross-protection as well as maintenance of energetic homeostasis both under wet or dry climatic conditions. We may suggest that plastic-induced changes in stress resistance traits and energy metabolites in *D. ananassae* are likely to counter future drier conditions expected due to climate change.

## MATERIAL AND METHODS

### Collection and cultures

Wild-caught *Drosophila* species were collected during two seasons, wet or rainy (July and August) and dry or autumn (mid September to mid November), from five local sites but each one separated by ∼3 to 4 km in the university town Rohtak, India (latitude 28.08 °N, altitude 220 m) in the year 2015. The flies were collected during one week in the mid rainy or autumn season. Based on our past collection records, the relative abundance of *Drosophila ananassae* is ∼30% in rainy season and ∼20% in the autumn. Therefore, we collected 730 flies in rainy season and 416 flies in autumn season, which included different *Drosophila* species. However in the laboratory, assortment of wild-caught flies provided 192 flies of *D. ananassae* in rainy season and 136 in autumn season. For each season, flies were used to set up three replicate populations in 300 ml culture bottles, each with 40 pairs of *D. ananassae*. Further, adult flies of each bottle were allowed to oviposit on cornmeal-yeast-agar medium in four culture bottles in order to maximize the number of laboratory-raised flies.

The wet season wild-caught flies were reared under wet season-specific simulated conditions (25±1°C and 78±2% RH). The resulting adult flies of G_1_ and G_2_ were used for assessment of basal, acclimated, cross-tolerance effects of three stressors (heat, drought and starvation) along with simultaneous analysis of control or unacclimated flies. For wet season, one-week-old flies of G_1_ and G_2_ generations were analyzed for changes in three stress-related traits and energy metabolites (body lipids, proline and trehalose). All experiments on wet season flies were completed before the onset of autumn season. Likewise, for autumn or dry season, collection of wild flies, setting up of mass populations in triplicate and rearing under dry conditions (25±1°C; 40±2% RH) were conducted during the autumn season. For dry season flies, stress resistance traits (heat knockdown, desiccation or starvation resistance) as well as energy metabolites (trehalose, proline and total body lipids contents), were assessed in three replicates of thirty flies. Control experiments were run simultaneously.

### Stress resistance assays

Firstly, heat knockdown time was measured in three replicates of thirty flies of both the seasons. Individual males and females were placed into 5 ml glass vials submerged into a water bath at a constant temperature of 37°C. Flies were scored for knockdown time (in minutes and seconds). Secondly, desiccation resistance was measured as the time to dehydration effect under dry air (∼8% RH) in flies of both the sexes of wet or dry season. Each vial contained 2 g of silica gel at the bottom overlain with a foam disc to avoid contact with flies. We placed ten flies in such plastic vials (40×100 mm) in which the open end was covered with muslin cloth. Finally, such vials were kept in the desiccator chamber (Secador electronic desiccator cabinet; www.tarsons.in) which maintained ∼8% relative humidity. Number of immobile flies was counted after hourly intervals; and LT_100_ values were recorded. Finally, for three replicates of thirty flies, starvation resistance was measured as survival time until death under humid conditions (∼90% RH), but without food. Ten adult flies were placed in a dry plastic vial which contained foam sponge impregnated with 8 ml of water and 2 mg sodium benzoate (to prevent any bacterial growth). The mortality time was recorded twice a day until all flies died from starvation.

### Assessment of direct and cross-tolerance effects

Direct as well as cross-tolerance effects were assessed for each stressor (heat, drought or starvation) in *D. ananassae* flies of wet or dry seasons. For each stressor, different groups (three replicates of thirty flies each) of acclimated flies were tested for changes in other two stress-related traits. For three stressors (heat, desiccation or starvation), we tested nine acclimation-by-test combinations (i.e. three direct effects and six cross tolerance effects). For analysis of cross-tolerance, acclimated/hardened fly groups were tested for changes (increase/decrease/no effect) in the level of each stress-resistance trait. Therefore, for testing direct and cross tolerance due to (a) heat hardening, three groups of thirty flies of both the sexes were subjected to 2 h heat stress followed by 2 h recovery period on nutrient agar medium; and thereafter flies were analyzed for change in heat knockdown, desiccation or starvation resistance. (b) Desiccation acclimation was given to flies for 4 h followed by 4 h recovery period; and these flies were analyzed for heat knockdown, desiccation and starvation resistance. (c) Flies were exposed to 20 h for starvation acclimation followed by 15 h recovery period; followed by testing for changes in heat knockdown, starvation and desiccation resistance.

### Estimation of energy metabolites

Body lipid content was estimated on G_1_ or G_2_ flies (three replicates of 30 flies of each season and sex) reared under wet or dry season-specific conditions. For lipid content individual flies were dried in 2 ml Eppendorf tubes (www.tarsons.in) at 60°C for 48 h and then weighed on Sartorius microbalance (Model-CPA26P; 0.001 mg precision; www.sartorious.com). Thereafter, 1.5 ml di-ethyl ether was added in each Eppendorf tube and kept for 24 h under continuous shaking (200 rpm) at 37°C. Finally, the solvent was removed and individuals were again dried at 60°C for 24 h and reweighed. Lipid content was calculated per individual by subtracting the lipid-free dry mass from initial dry mass per fly.

For trehalose content estimation, each of the three replicates of 30 flies of each season and sex were homogenized (Labsonic@ M; www.sartorious.com) with 300 μl Na2CO3 and incubated at 95°C for 2 h to denature proteins. An aqueous solution of 150 μl acetic acid (1 M) and 600 μl sodium acetate (0.2 M) was mixed with the homogenate. Thereafter, the homogenate was centrifuged (Fresco 21, Thermo-Fisher Scientific, Pittsburgh, USA) at 12,000 rpm (9660×***g***) for 10 min. For trehalose estimation, aliquots (200 μl) were placed in two different tubes; one was taken as a blank whereas the other was digested with trehalase at 37°C using the Megazyme trehalose assay kit (K-Treh 10/10, www.megazyme.com). In this assay, released D-glucose was phosphorylated by hexokinase and ATP to glucose-6-phosphate and ADP, which was further coupled with glucose-6-phosphate dehydrogenase and resulted in the reduction of nicotinamide adenine dinucleotide (NAD). The absorbance by NADH was measured at 630 nm (UV-2450-VIS, Shimadzu Scientific Instruments, Columbia, USA). The pre-existing glucose level in the sample was determined in a control reaction lacking trehalase and subtracted from total glucose concentration.

Proline content was estimated in each of the three replicates of 30 flies of each season and sex. Proline concentrations in fly homogenates were determined by the modified method after Bergman and Loxley (1970). In this assay, interference from primary amino acids gets eliminated by nitrous acid treatment and the excess nitrous acid is removed by heating with ammonium chloride followed by hydrochloric acid. Interfering materials are also removed by absorption to the protein-sulphosalicylic acid complex.

Thirty adult flies were homogenized in 3 ml of sulphosalicylic acid. Following centrifugation, 50 μl of the homogenate was added to 15 μl of freshly prepared 1.25 M sodium nitrite solution and content were mixed and kept at room temperature for 20 min. Further, 15 μl of 1.25 M ammoniumchloride solution was added and content was mixed followed by addition of 60 μl of concentrated hydrochloric acid. The content were mixed and heated in a boiling water bath for 20 min. The tubes were cooled and 60 μl of 10 N sodium hydroxide was added. To the resulting solution, we added 200 μl glacial acetic acid and 200 μl of ninhydrin solution in each capped tube. The solutions were mixed and incubated for 60 min at 100°C. Following incubation, the samples were extracted with toluene, and absorbance of the aqueous phase was quantified spectro-photometrically at 520 nm and the amount of proline was estimated in reference to a standard curve.

### Analysis of rate of change in energy metabolites after hardening/acclimation pre-treatments

We conducted independent experiments for each stressor to find change in the rate of accumulation or utilization of three energy metabolites (trehalose, proline and body lipids). Such changes were assessed in three replicates of 30 flies of each season as well as sex as a function of different time durations of hardening/acclimation by a stressor. Independent groups of flies were (a) heat hardened for 1, 2, 3, 4 and 5 h at 34°C with 2 h recovery; (b) desiccation acclimation for 1, 2, 3, 4 and 5 h at 8% relative humidity with 4 h recovery; (c) starvation acclimation for 10, 15, 20, 25 and 30 h followed by 15 h recovery; and these respective flies were tested for changes in the level of each of three energy metabolites.

### Assessment of energy metabolites mediated cross-protection

For acclimated and non-acclimated (control flies), changes in three energy metabolites (trehalose, body lipids and proline content) due to each stressor were measured in three replicates each of thirty flies. This was done for flies reared under season-specific wet or dry conditions. For such analysis, we used data on flies after (a) heat hardening (2 h heat stress followed by 2 h recovery); (b) desiccation (5 h acclimation followed by 4 h recovery); (c) starvation (25 h starvation acclimation followed by 15 h recovery). Finally, percent change in accumulation or utilization of three energy metabolites due each stressor were calculated to find possible cross-protection between multiple stressors.

### Treatment and analysis of data

Data for heat knockdown time at 37°C (in minutes and seconds) were recorded on individual male and female flies of *D. ananassae* reared under wet or dry season-specific simulated conditions. For the other two stressors (desiccation or starvation), we recorded survival mortality of 10 flies per vial as a function of shorter (at hourly) for desiccation; and twice daily (08:00 h and 20:00 h) for starvation resistance until all the flies died. For each season and sex (three replicates of 30 flies) data on basal level (or control) and hardened/acclimated flies were represented as mean±s.e.m. (Table 1) while effects of treatments and sex were calculated on the basis of ANOVA (Tables S1 and S2). Seasonal differences in stress-related traits were compared with *t*-test and in terms of fold differences (Table 1).

The data on assays for three stressors (heat, drought or starvation) were used for calculating absolute acclimation/hardening capacity (AAC) (i.e. difference in trait values between acclimated flies/control flies) following Kellett et al. (2005). Further, we also calculated RAC (i.e. absolute acclimation capacity divided by control value of unacclimated flies). For all the three stress-related traits, we represented AAC in the form of bars while RAC values were depicted on the top of each bar. Illustrations depicted simultaneous comparison of direct acclimation effect and cross tolerance effects for female flies of wet or dry season (Fig. 1). For heat resistance, correlation between direct heat-hardening effects and cross-tolerance effects due to desiccation acclimation were represented (with box-and-whisker plots) for flies of two seasons as well as sexes (Fig. 2). Further, in accordance with the objectives of this study, data on each of the three energy metabolites (µg mg^−1^ fly^−1^) in control flies were compared with heat hardened or flies acclimated to desiccation and starvation; and percent changes (+ or −) were calculated to compare acclimation effects across seasons and sexes (Table 2).

Data obtained from independent sets of experiments on rate of metabolite change as a consequence of different durations (1, 2, 3, 4 or 5 h) for heat hardening or desiccation acclimation were subjected to regression analysis for calculation of regression slope values (Table 3), and seasonal differences in slope values were compared with *t*-test. Finally, season-specific differences in the accumulation and utilization (calculated in terms of percent increase or decrease) of three energy metabolites (body lipids, trehalose and proline) due to either heat hardening or acclimation to desiccation or starvation were schematically represented to highlight possible energy metabolite-mediated cross-protection in wet or dry season female flies (Fig. 3). For stressor acclimated/hardened flies, the energy content (trehalose, proline and body lipids) was calculated using standard conversion factors (Schmidt-Nielsen, 1990). Statistical calculations and illustrations were made with the help of Statistica 5.0 and Statistica 7 (Statsoft).

## Supplementary Material

Supplementary information
