# Supplementary material for: Tropical Drosophila ananassae of wet-dry seasons show cross resistance to heat, drought and starvation
Source: Biol Open. 2017 Nov 15;6(11):1698–706. doi: 10.1242/bio.029728 (PMC5703618; doi:10.1242/bio.029728)
Supplement: Supplementary information [file biolopen-6-029728-s1.pdf]

**Table S1.** Results of ANOVA for treatment groups (control vs acclimated) and due to two seasons as well as sexes for three stress related traits in *D. ananassae* reared under wet or dry season specific simulated condition.

| | | Heat knockdown | | Desiccation resistance | | Starvation resistance | |
|---|---|---|---|---|---|---|---|
| | df | MS | F | MS | F | MS | F |
| Sn | 1 | 20869.35 | 4989.72*** | 1122.338 | 1891.64*** | 54481.07 | 5230.33*** |
| T | 1 | 37200.60 | 8894.41*** | 701.784 | 1182.82*** | 29260.42 | 2809.08*** |
| S | 1 | 17069.70 | 4081.09*** | 487.920 | 822.37*** | 13053.75 | 1253.19*** |
| Sn × T | 1 | 160.07 | 38.27*** | 5.221 | 8.80** | 15714.02 | 15.08.59*** |
| Sn × S | 1 | 1622.40 | 387.91*** | 4.48.3 | 7.56** | 4558.82 | 437.66*** |
| T × S | 1 | 2172.02 | 519.31*** | 28.428 | 47.914*** | 1926.67 | 184.97*** |
| Sn × T × S | 1 | 4183.35 | 1000.21*** | 3.361 | 5.664* | 2693.40 | 258.57*** |
| Error | 232 | 4.18 | | 0.593 | | 10.42 | |

Sn = Season; T = Treatment; S = Sex; ****p* <0.001; ***p* <0.01; **p*<0.5

Biology Open • Supplementary information

**Table S2.** Results of ANOVA for treatment groups (control vs acclimated) and due to two seasons as well as sexes for three energy metabolites in *D. ananassae* reared under wet or dry season specific simulated condition.

| | | Trehalose | | Proline | | Total body lipids | |
|---|---|---|---|---|---|---|---|
| | df | MS | F | MS | F | MS | F |
| Sn | 1 | 8044.35 | 10800.51*** | 150016.6 | 296127.2*** | 467625.7 | 80.611*** |
| S | 1 | 232.04 | 311.54*** | 2076.2 | 4098.4*** | 218282.7 | 37.628*** |
| T | 3 | 7342.36 | 9858.00*** | 14674.9 | 28967.7*** | 375481.4 | 64.727*** |
| Sn × T | 3 | 2522.78 | 3387.13*** | 123.3 | 243.4*** | 87318.1 | 15.052*** |
| Sn × S | 1 | 1101.22 | 1478.52*** | 15360.3 | 30320.6*** | 68066.6 | 11.733*** |
| T × S | 3 | 148.15 | 198.91*** | 6.90 | 13.7*** | 14502.1 | 2.499** |
| Sn × T × S | 3 | 174.54 | 234.35*** | 33.50 | 66.2*** | 11547.5 | 1.99* |
| Error | 464 | 0.744 | | 0.507 | | 5800.98 | |

Sn = Season; T = Treatment; S = Sex; ****p* <0.001; ***p* <0.01; **p*<0.5; Unit= (µg $mg^{-1}$ $fly^{-1}$)

Biology Open • Supplementary information
